# Peripheral arterial stiffness during electrocutaneous stimulation is positively correlated with pain-related brain activity and subjective pain intensity: an fMRI study

**DOI:** 10.1038/s41598-021-83833-6

**Published:** 2021-02-24

**Authors:** Toshio Tsuji, Fumiya Arikuni, Takafumi Sasaoka, Shin Suyama, Takashi Akiyoshi, Zu Soh, Harutoyo Hirano, Ryuji Nakamura, Noboru Saeki, Masashi Kawamoto, Masao Yoshizumi, Atsuo Yoshino, Shigeto Yamawaki

**Affiliations:** 1 Department of System Cybernetics, Graduate School of Engineering, Hiroshima University, 1-4-1 Higashi-Hiroshima, Hiroshima, 739-8527 Japan; 2 Center for Brain, Mind and KANSEI Sciences Research, Hiroshima University, 1-2-3 Kasumi, Minami-ku, Hiroshima, 734-8551 Japan; 3 College of Engineering, Academic Institute, Shizuoka University, 3-5-1, Johoku, Nakaku, Hamamatsu, 432-8561 Japan; 4 Department of Anesthesiology and Critical Care, Graduate School of Biomedical and Health Sciences, Hiroshima University, 1-2-3 Kasumi, Minami-ku, Hiroshima, 734-8551 Japan; 5 Department of Cardiovascular Physiology and Medicine, Graduate School of Biomedical and Health Sciences, Hiroshima University, 1-2-3 Kasumi, Minami-ku, Hiroshima, 734-8553 Japan; 6 Department of Psychiatry and Neurosciences, Graduate School of Biomedical and Health Sciences, Hiroshima University, 1-2-3 Kasumi, Minami-ku, Hiroshima, 734-8551 Japan

**Keywords:** Brain imaging, Pain

## Abstract

Brain activity associated with pain perception has been revealed by numerous PET and fMRI studies over the past few decades. These findings helped to establish the concept of the pain matrix, which is the distributed brain networks that demonstrate pain-specific cortical activities. We previously found that peripheral arterial stiffness ${\beta }_{\text{art}}$ responds to pain intensity, which is estimated from electrocardiography, continuous sphygmomanometer, and photo-plethysmography. However, it remains unclear whether and to what extent ${\beta }_{\text{art}}$ aligns with pain matrix brain activity. In this fMRI study, 22 participants received different intensities of pain stimuli. We identified brain regions in which the blood oxygen level-dependent signal covaried with ${\beta }_{\text{art}}$ using parametric modulation analysis. Among the identified brain regions, the lateral and medial prefrontal cortex and ventral and dorsal anterior cingulate cortex were consistent with the pain matrix. We found moderate correlations between the average activities in these regions and ${\beta }_{\text{art}}$ (*r* = 0.47, *p* < 0.001). ${\beta }_{\text{art}}$ was also significantly correlated with self-reported pain intensity (*r* = 0.44, *p* < 0.001) and applied pain intensity (*r* = 0.43, *p* < 0.001). Our results indicate that ${\beta }_{\text{art}}$ is positively correlated with pain-related brain activity and subjective pain intensity. This study may thus represent a basis for adopting peripheral arterial stiffness as an objective pain evaluation metric.

## Introduction

Pain perception is an important signal that communicates actual or potential tissue damage^1^. Because pain is an individual experience, its evaluation primarily relies on self-reported verbal descriptions^2^ and subjective rating metrics, such as the visual analogue scale (VAS)^3^ and numeric rating scale (NRS)^4^. However, it is difficult to accurately describe pain perception using subjective measures such as these, and ratings largely depend on individual experience and characteristics. This problem is critical for patients with dementia, young children^5^, or those under general anaesthesia, for example. Thus, objective and quantitative pain metrics for capturing pain perception are needed in such cases, and should be developed based on insights gained from physiological responses to the pain.

Pain perception starts from the stimulation of nociceptors followed by activation of Aδ or C nerve fibres. The signal is then transmitted to the dorsal horn of the spinal cord via peripheral nerves, and ascends through the lateral spinothalamic tract to the thalamic nuclei. At the same time, peripheral sympathetic nerves are activated and acutely contract the peripheral artery. Finally, the information reaches the somatosensory cortex to produce physical nociception^5,6^, which can evoke voluntary avoidance of the noxious stimulus and an involuntary physiological response via the spinal reflex or autonomic nervous system. Pain perception thus involves both the autonomic nervous system and higher brain functions. For this reason, indirect measures of autonomic nervous system responses, including heart rate, blood pressure, and skin electrical conductance, have been used as biomarkers of pain^7^. However, the relationship between peripheral sympathetic activity and brain activity in response to pain has not yet been fully investigated.

Previous functional magnetic resonance imaging (fMRI) studies on the neuronal processing of pain perception have successfully identified pain-related brain areas. The corresponding areas are collectively known as the pain matrix, and typically include the primary and secondary somatosensory cortices (SI and SII), anterior cingulate cortex (ACC), lateral prefrontal cortex (LPFC), medial prefrontal cortex (MPFC), insula, supplementary motor area (SMA), and thalamus^6,8,9^. Moreover, an fMRI-based measure derived from machine learning analysis has been found to successfully predict pain intensity^10^. This also indicates that activity evoked in the pain matrix is strongly associated with the sensation of pain.

In recent work, we proposed a mathematical model to estimate peripheral arterial stiffness that reflects the peripheral sympathetic nerve activity from electrocardiography, continuous sphygmomanometry, and photo-plethysmography^11^. We then derived a peripheral arterial stiffness-based metric to evaluate pain intensity and qualities^12^, and found that peripheral arterial stiffness could predict pain perception^13^. Although it is plausible to assume that peripheral arterial stiffness is correlated with brain activity in pain-related areas, no studies have explored this.

The aim of this study was to explore the correlation between peripheral arterial stiffness and brain activity in response to pain stimuli. We first proposed an approximated mathematical model to estimate peripheral arterial stiffness in an fMRI environment, because some of the measurement instruments of the previous model^12,13^ were not fMRI-compatible. To test the estimation accuracy of the proposed model, we first conducted a measurement experiment outside the fMRI environment (Experiment 1) and estimated the peripheral arterial stiffness simultaneously using both the proposed model and the previous model^12,13^. We then conducted an fMRI experiment while applying electrocutaneous stimuli to participants (Experiment 2) and identified brain regions in which the blood oxygen level-dependent (BOLD) signal covaried with the ${\beta }_{\text{art}}$ response. Focussing on regions that were consistent with the pain matrix, we performed a correlation analysis between the activities in these regions and the estimated arterial stiffness, and subjectively rated pain intensity.

## Methods

### Approximated peripheral arterial stiffness ${\upbeta }_{\text{art}}$ estimation method in an fMRI environment

We previously proposed a model, called the log-linearised peripheral arterial viscoelastic model, that can evaluate peripheral sympathetic nerve activity by estimating arterial stiffness (${\beta }_{\text{art}}^{^{\prime}}$) using cardiac cycles, continuous arterial pressure (${P}_{b}\left(t\right)$), and arterial wall diameter (${P}_{l}\left(t\right)$)^13^. This model has been adopted to support sympathetic nerve activity evaluation in real-time in endoscopic thoracic sympathectomy^14^ when the patient is under general anaesthesia. The cardiac cycles are obtained from electrocardiography, and ${P}_{b}\left(t\right)$ and ${P}_{l}\left(t\right)$ are estimated using continuous sphygmomanometer and photo-plethysmography, respectively. The model is defined by the following equation:

1 $${P}_{b}\left(t\right)=\mu {\ddot{P}}_{l}\left(t\right)+\eta \dot{{P}_{l}}\left(t\right)+\text{exp}\left\{{\beta }_{art}^{^{\prime}}{P}_{l}\left(t\right)+{P}_{b{\beta }_{art0}^{^{\prime}}}+{P}_{b{\beta }_{artnl}^{^{\prime}}}\left({P}_{l}\left(t\right)\right)\right\}$$

where $\mu$ is the inertia, $\eta$ is the viscosity, ${\beta }_{art}^{^{\prime}}$ is the peripheral arterial stiffness, ${P}_{b{\beta }_{art0}^{^{\prime}}}$ is the constant pressure component, and ${P}_{b{\beta }_{artnl}^{^{\prime}}}\left({P}_{l}\left(t\right)\right)$ is the nonlinear stiffness pressure component originating in the vein. In addition, *t* represents time and the dot operator on ${P}_{l}\left(t\right)$ represents the time derivative. This model is applicable to cases where ${P}_{b}\left(t\right)$ and ${P}_{l}\left(t\right)$ are available, but no fMRI-compatible device allows the measurement of ${P}_{b}\left(t\right)$. We instead used fMRI-compatible sphygmomanometer to measure systolic blood pressure (${P}_{\text{SYS}}$) and diastolic blood pressure (${P}_{\text{dia}}$) on a beat-to beat basis. Therefore, we approximated the peripheral arterial stiffness^15^ using the measurable parameters. Please refer to Supplemental Material S2 for the approximation process. The proposed model is given by the following equation:

2 $${\beta }_{\text{art}}=\frac{\text{ln}\left(\frac{{P}_{\text{SYS}}}{{P}_{\text{dia}}}\right)}{{P}_{\text{lmax}}-{P}_{\text{lmin}}}$$

where ${P}_{\text{lmax}}$ and ${P}_{\text{lmin}}$ are the maximum and minimum values of the photo-plethysmogram within a heartbeat. The model focuses on the linearly approximated relationship between ${P}_{b}\left(t\right)$ and ${P}_{l}\left(t\right)$, where ${\beta }_{\text{art}}$ is its slope. Using this method, ${\beta }_{\text{art}}$ can be calculated for each heartbeat. Here, the measured electrocardiogram (ECG) was used to determine the R-R interval for extracting ${P}_{\text{SYS}}$, ${P}_{\text{dia}}$, ${P}_{\text{lmax}}$, and ${P}_{\text{lmin}}$ per heartbeat.

**Figure 1:**
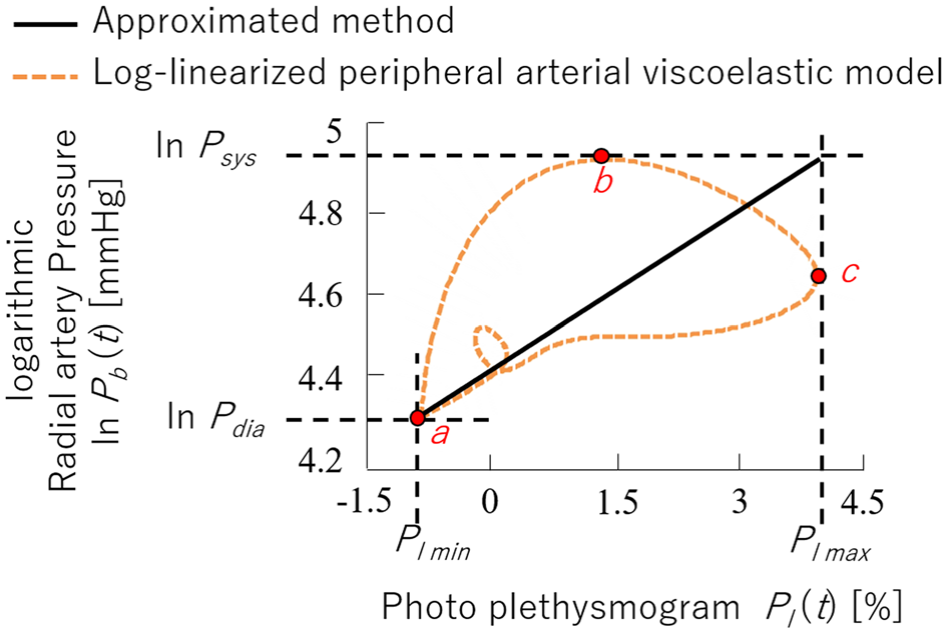
Example of a measured Lissajous curve between radial artery pressure and the associated photo-plethysmography data. The previously used log-linearised peripheral arterial viscoelastic model estimates peripheral arterial stiffness (${\beta }_{art}^{^{\prime}}$) by fitting the model to a Lissajous curve of the photo-plethysmogram (${P}_{l}\left(t\right)$) and arterial pressure (${P}_{b}\left(t\right)$), as represented by the dashed curve. However, the model proposed in this paper linearly approximates the relationship between ${P}_{l}\left(t\right)$ and ${P}_{b}\left(t\right)$, as represented by the solid black line, so that ${\beta }_{art}$ corresponds to the slope. *a* denotes the point at which the photo-plethysmogram and arterial pressure are at their minimum (*P*_*l*_(*t*) = *P*_*l min*_, *P*_*b*_(*t*) = *P*_*dia*_). *b* denotes the point at which the arterial pressure is at its maximum (*P*_*b*_(*t*) = *P*_*sys*_). *c* denotes the point at which the arterial volume is at its maximum (*P*_*l*_(*t*) = *P*_*l max*_). The solid line represents the slope between points (ln *P*_*dia*_, *P*_*l min*_) and (ln *P*_*sys*_, *P*_*l max*_) corresponding to the peripheral arterial stiffness.

Figure 1 shows an example of the Lissajous curve between the radial artery pressure and photo-plethysmogram during a single heartbeat. First, we can assume that the photo-plethysmogram, *P*_*l*_(*t*), is proportional to the arterial volume. As depicted by point *a* in the figure, the arterial volume becomes minimum when the artery pressure is the lowest (*P*_*b*_(*t*) = *P*_*dia*_). The arterial volume increases with a phase delay during the increase of the arterial pressure from diastolic pressure to systolic pressure (from point *a* to point *b*). Here, the increase rate depends on the arterial stiffness, and the phase delay of increase depends on the arterial inertia and viscosity. The arterial volume continues to increase to its maximum due to the phase delay, even after the arterial pressure reaches its maximum and, in turn, starts to decrease (from point *b* to point *c*). Finally, the arterial volume decreases and returns to its minimum (from point *c* to point *a*). The small circle that appears during this decrease is caused by the increase of arterial pressure due to a reflecting pulse wave. Equation 1 approximates the Lissajous curve and estimates ${\beta }_{art}^{^{\prime}}$, but the approximated method estimates ${\beta }_{art}$ using the linear regression of two points, (${P}_{\text{lmax}}$, ln ${P}_{\text{SYS}}$) and (${P}_{\text{lmin}}$, ln ${P}_{\text{dia}}$), which allows peripheral arterial stiffness to be estimated using the present fMRI-compatible devices. Hereafter, we denote ${\beta }_{\text{art}}$ as the arterial stiffness estimated by the proposed model (Eq. (2) and ${\beta }_{\text{art}}^{^{\prime}}$ as that estimated by the previous model (Eq. (1)).

### Experiment 1: Verification of the estimation accuracy of the proposed model

In accordance with the Declaration of Helsinki, written informed consent was obtained from all participants before the experiments were performed. The experimental protocols were approved by the Research Ethics Committee of Hiroshima University and Mazda Motor Corporation (approval numbers E-965-5, E-17 and TRC-152-6). Thirteen healthy men (mean age ± standard deviation: 23.5 ± 1.4 years) participated in the experiment.

**Figure 2:**
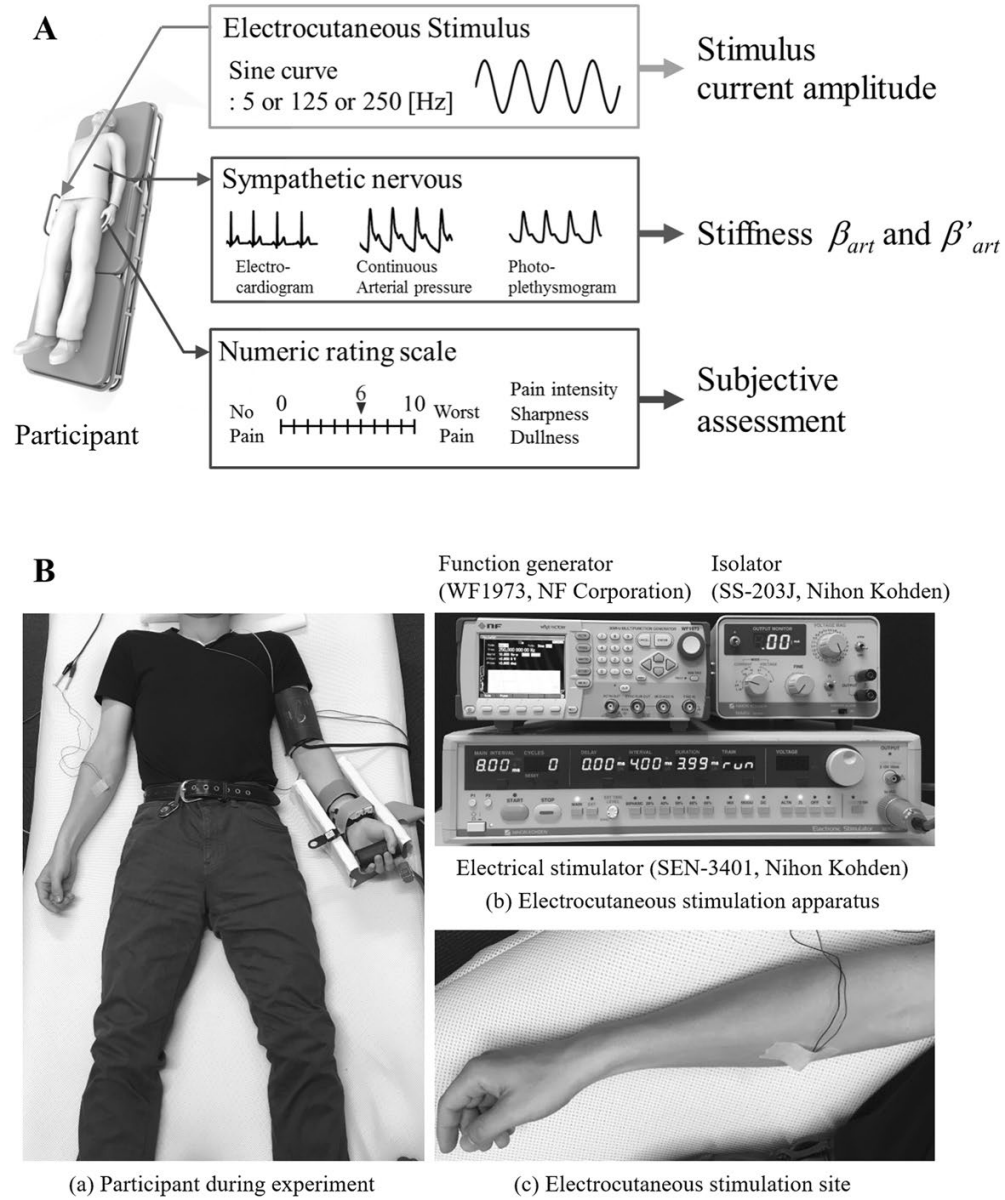
Experimental environment. (**A**) Measurements of biosignals and the stimuli applied. Sinusoidal electrocutaneous stimuli with different amplitudes and frequency conditions were applied to evoke different pain intensities. Peripheral arterial stiffness was calculated from electrocardiograms, continuous arterial pressures, and photo-plethysmography. Subjective assessments of pain intensity were obtained verbally. (**B**) (a) Shows a participant with sensors attached to measure electrocardiograms, continuous arterial pressure, and photo-plethysmography, and (b) shows the pain stimulation apparatus, which consisted of a function generator, an isolator, and an electrocutaneous stimulator.

We measured biological signals and obtained subjective pain assessments during the application of electrocutaneous nociceptive stimuli, as shown in Fig. 2A. To test the estimation accuracy of the proposed model, peripheral arterial stiffnesses ${\beta }_{\text{art}}^{^{\prime}}$ and ${\beta }_{\text{art}}$ were then estimated by the previous model (Eq. (1) and the proposed model (Eq. (2)), respectively, based on the measured biological signals (electrocardiogram, sphygmomanom, and photo-plethysmogram).

Figure 2B shows the experimental configuration and measurement apparatus. Participants reclined in a supine position. Electrocardiogram signals were measured with a 3-lead electrocardiograph, and left radial arterial blood pressure was measured using a non-invasive biological information monitor (BP-608 Evolution II CS, Omron Colin, Kyoto, Japan). Photo-plethysmography was conducted using a pulse oximeter attached on the left index finger of the participant (OLV-3100, Nihon Kohden, Tokyo, Japan). These data were collected at a sampling rate of 1000 Hz and stored on a computer using an analogue/digital converter (CSI-360116, Interface, Hiroshima, Japan).

During the experiment, participants received sine-wave electrocutaneous stimulation at 5, 125, and 250 Hz. The electrocutaneous stimulation currents were generated by an electrocutaneous stimulator (SEN-3401, Nihon Kohden, Tokyo, Japan), an isolator (SS-203J, Nihon Kohden, Tokyo, Japan), and a function generator (WF1973, NF, Tokyo, Japan). The stimuli were applied to the skin surface on the medial side of the right forearm through an electrode (NM-990W, Nihon Kohden, Tokyo, Japan) (Fig. 2B). Before applying stimulation, the skin was wiped with ethanol to increase skin conductance. It is worth noting that the electrocutaneous stimulator has a feedback control system that stabilises the applied current to ensure that the desired current is applied across different levels of skin conductance.

Considering that pain thresholds differ substantially between individuals, stimulation currents were configured before this experiment by applying stimulation currents with different amplitudes and determining a standard amplitude at which participants rated the pain intensity as 3 out of 10 on the NRS. Specifically, first, we explained the NRS-based rating criteria to participants, where 0 indicates no pain and 10 indicates the worst imaginable pain. We then applied a stimulation current of about 0.1 mA and asked the participant to rate the pain experienced. Subsequently, the stimulation current was progressively increased, typically by 0.02 mA per trial, until the participant rated the pain as 3 out of 10. The applied current typically reached about 0.15 to 0.18 mA. This calibration was carried out before the experiment began, when the participant reclined in a supine position inside the MRI bore with all sensors and stimulus electrodes attached.

The experiments were then conducted using the following protocol: In a single trial, we sequentially performed 24 s of the continuous electrocutaneous stimulation task, 20 s of self-reported pain evaluation, and 20 s of rest. There were seven trials with different continuous electrocutaneous stimulation conditions, in which current amplitudes were applied to participants in the order of 1.5, 1.0, 0.5, 0, 0.5, 1.0, and 1.5 times the standard amplitude. Finally, to test the estimation accuracy of the proposed model, a linear correlation analysis was performed on the peripheral arterial stiffnesses ${\beta }_{art}$ and ${\beta }_{art}^{^{\prime}}$ estimated from Eq. (2) and Eq. (1), respectively. A correlation with a *p*-value < 0.05 was considered significant.

### Experiment 2: Analysis of the relationships between ${\upbeta }_{\text{art}}$, brain activity, self-reported pain intensity, and stimulation amplitude

**Figure 3:**
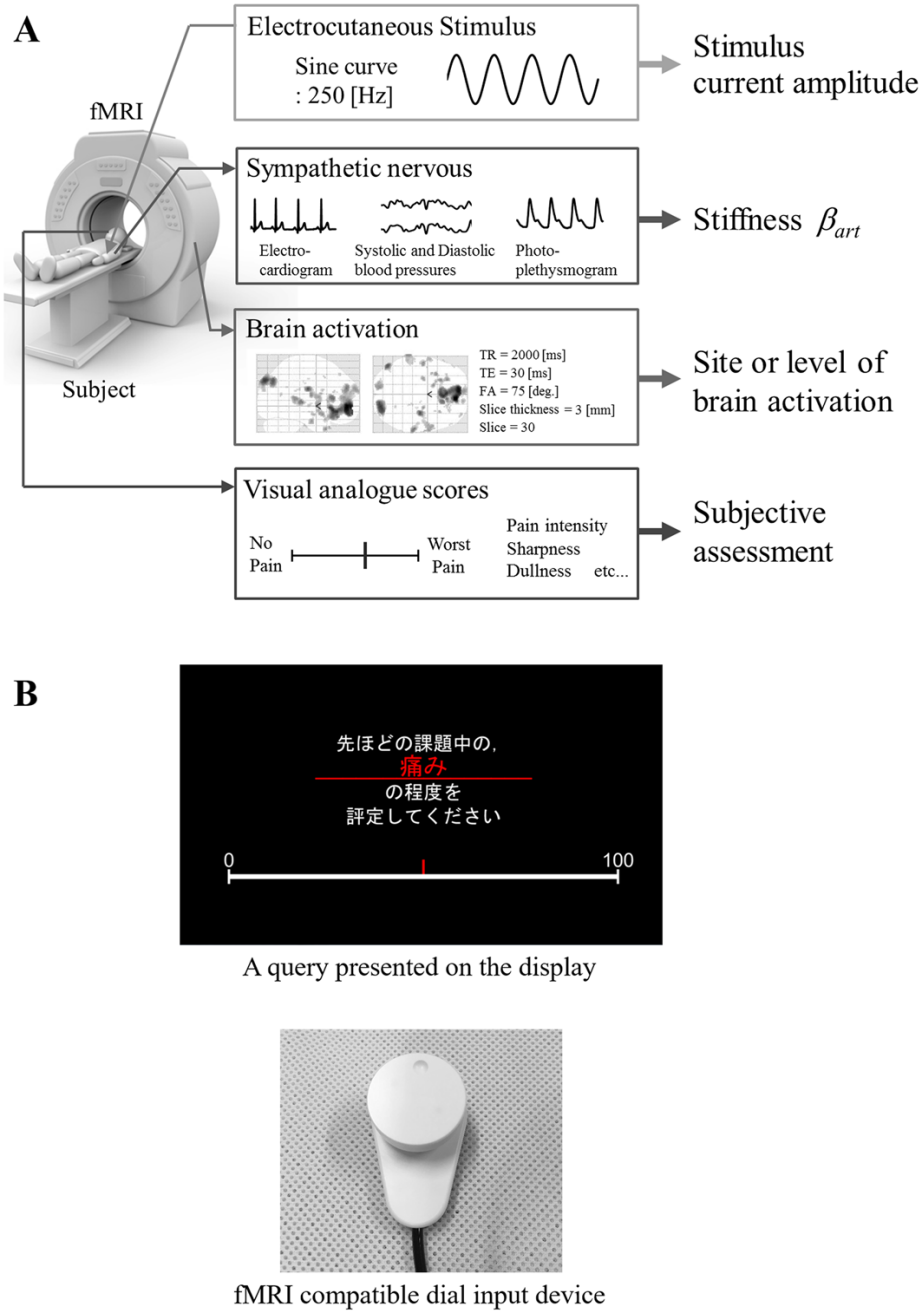
Overview of the experiment comparing peripheral arterial stiffness (${\beta }_{art}$), brain activity (contrast estimates), and subjective pain assessment during electrocutaneous stimulation. (**A**) Electrocutaneous stimuli were applied during functional magnetic resonance imaging (fMRI) acquisition, and fMRI-compatible devices were simultaneously used to record electrocardiograms, blood pressure, and photo-plethysmography. ${\beta }_{art}$ was calculated using the proposed model (Eq. (2). (**B**) Devices for measuring pain intensity were as follows: (a) Shows the display for presenting instructions and the visual analogue scale (VAS). In this example, the instruction reads “Please rate the intensity of pain you experienced in the previous task.” Participants moved the red vertical bar of the VAS to indicate their response; (b) shows the dial device for pain intensity input on the VAS.

We then analysed the relationships between peripheral sympathetic nerve activity represented by ${\beta }_{art}$, brain activity, and self-reported pain intensity. To this end, we concurrently measured the biological signals, which were used to calculate ${\beta }_{art}$, and fMRI brain activity during electrocutaneous stimulation. Figure 3A shows an overview of the experiment. Twenty-two healthy men (mean age ± standard deviation: 22.7 ± 1.0 years) participated in this experiment. Of these participants, six had also participated in Experiment 1.

Electrocardiogram signals from a three-lead electrocardiograph and a photoplethysmograph attached to the left index finger were measured using Biopac MP 150 system (Biopac Systems, Goleta, CA). *P*_SYS_ and *P*_dia_ (see Eq. (2)) during a single heartbeat were measured using the Care Taker module of Biopac Systems. The four types of time series variables mentioned above were measured at a sampling rate of 1000 Hz. Sine-wave electrocutaneous stimulation was applied at 250 Hz, and electrocutaneous stimulation currents were generated with an electrocutaneous stimulator (SEN-3401, Nihon Kohden), an isolator (SS-203J, Nihon Kohden, Tokyo, Japan), and a function generator (WF1973, NF). The stimulation was applied to the skin surface on the medial side of the right forearm through an electrode (NM-990W, Nihon Kohden Corp., Japan). We performed a pre-test to determine the standard current amplitude as in the previous experiment.

A single trial comprised a sequential continuous electrocutaneous stimulation task (8 s), self-reported pain evaluation (30 s), and rest (24 s). Each session comprised seven trials with different electrocutaneous stimulation conditions, whereby the amplitudes were configured in the same manner as for Experiment 1 based on the standard current amplitude. Participants completed three sessions. Hereafter, we denote the electrocutaneous stimulation condition with *s* times the standard current amplitude simply as *s* × stimulation. Prior to each session, participants were told what stimulation intensity they would receive and in what order to reduce the effects of anxiety on sympathetic nerve activity and overall brain activity. After each presentation of electrocutaneous stimuli, the VAS was presented on the MRI-compatible liquid crystal display (Fig. 3B(a)). Participants viewed the display through the mirror attached to the head coil, and were asked to subjectively report their pain by controlling a cursor (a red vertical bar) using an fMRI-compatible dial input device (Current Designs Inc., Philadelphia, PA, USA) (Fig. 3B(b)).

Brain activity during the session was measured using a 3.0 T MRI scanner (Siemens Magnetom Verio). Functional images were measured using the gradient echo sequence^16^. The acquisition parameters were 2000-ms repetition time (TR), 30-ms echo time (TE), 75-degree flip angle (FA), 3-mm slice thickness (without a gap), 3- × 3- × 4-mm voxel size, 30 slices, and 192-mm field of view (FOV). Structural images were measured using T1-weighted 3D magnetisation-prepared rapid gradient-echo imaging (MP-RAGE)^17^. The acquisition parameters were 2500-ms TR, 2.98-ms TE, 9-degree FA, 1-mm slice thickness, 1- × 1- × 1-mm voxel size, 176 slices, and 192-mm FOV.

Image processing and statistical analyses were performed using SPM12 software (Wellcome Department of Cognitive Neurology, www.fil.ion.ucl.ac.uk/spm). Details about the fMRI data analysis can be found in Supplemental Materials S2. As in previous research^18^, the first session was defined as a practice session and excluded from the final analysis to avoid the reported significant effects of learning. First, contrast estimates between the pain stimulus and rest duration were calculated to assess the whole-brain response to the pain stimulus. For each participant, the first-level individual analysis of the fMRI data was performed separately for sessions 2 and 3. The second-level group analysis was performed to identify clusters that had more than 20 adjoining active voxels with the peak activity threshold at an uncorrected *p*-value < 0.001.

In addition, a first-level individual parametric modulation analysis was conducted to identify regions in which hemodynamic activity covaried with arterial stiffness in response to the pain stimulus. Again, the analysis targets were the data obtained from sessions 2 and 3. The 0 × stimulus trials were excluded from the analysis to eliminate noise caused by false-positive or negative responses. The group-level analysis was performed using the same criteria as those used to calculate the contrast estimates. Among the identified brain regions, areas consistent with the pain matrix^6,8,9^ were extracted. The average contrast estimates for each stimulus duration within each extracted area were calculated for the voxels within a 3-mm radius centred on the peak coordinates. We then calculated the correlations between the contrast estimates, the maximum peripheral arterial stiffness ${\beta }_{art}$, and the VAS scores. In this correlation analysis, the contrast estimates, the arterial stiffnesses ${\beta }_{art}$, and VAS scores were standardised within each session for each participant. The VAS score was standardised to reduce the individual differences in the evaluation criteria^19,20^, and stiffness was standardised to reduce the individual differences in the light absorbance rate depending on the arterial radius measured by photoplethysmography. Contrast estimates were standardised to ensure consistency of data representation for comparisons with the other two indices. Correlations with a *p*-value < 0.001 were considered significant.

## Results

### Experiment 1: Verification of the estimation accuracy of the proposed model

**Figure 4:**
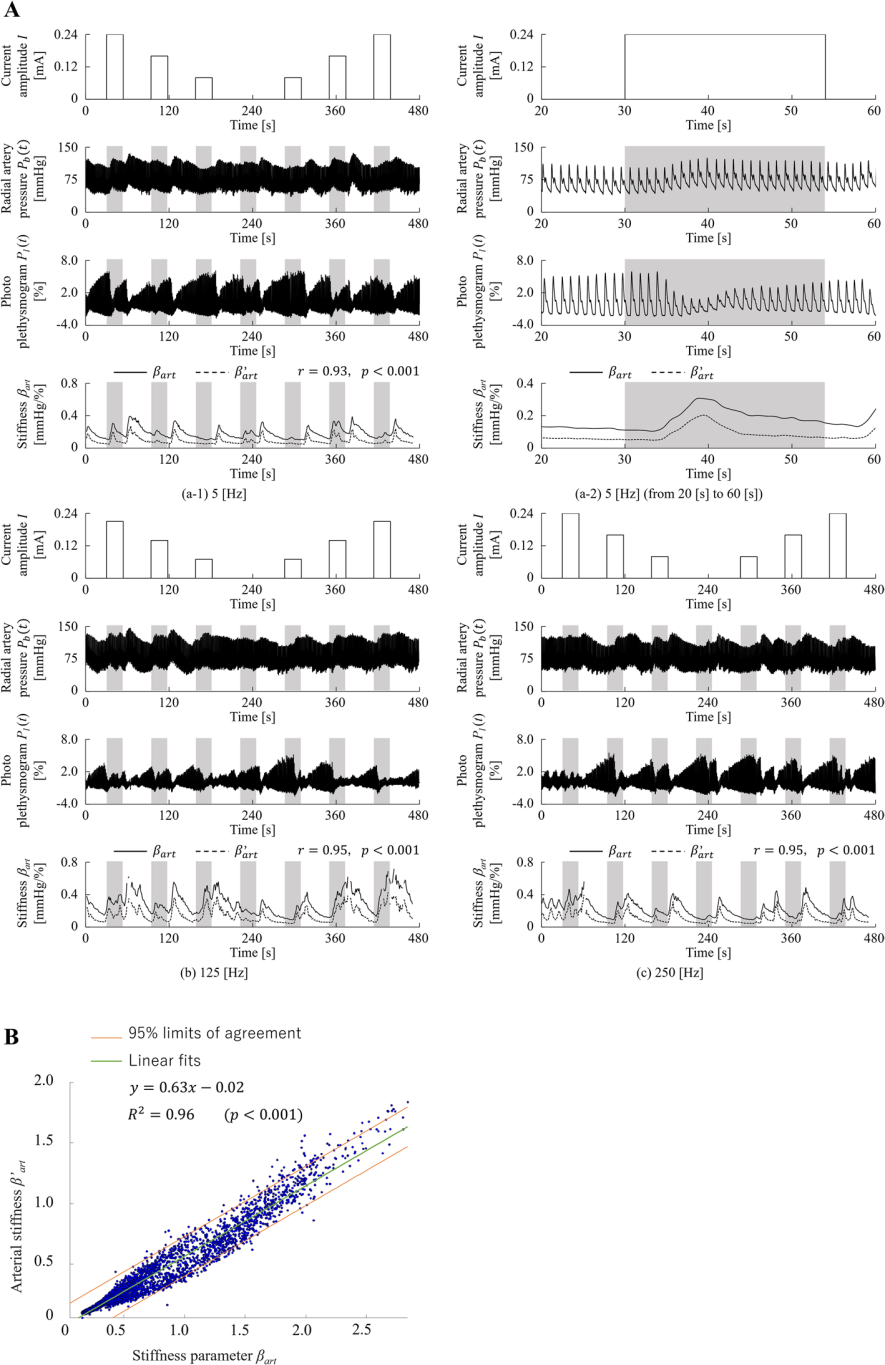
Verification of the estimation accuracy of the peripheral arterial stiffness of the proposed model. (**A**) From top to bottom, this panel shows amplitudes of electrocutaneous stimulations, radial arterial pressures, photo-plethysmography, stiffness parameter estimates (${\beta }_{art}$), and peripheral arterial stiffness (${\beta }_{art}^{^{\prime}}$) measured from Participant A. (a-1) and (a-2) show the measured results at a frequency of 5 Hz; (a-2) enlarges the time duration of the first trial from 20 to 60 s; (b) and (c) show the measured results at 125 Hz and 250 Hz, respectively. (**B**) Shows a comparison of the peripheral arterial stiffnesses ${\beta }_{art}$ and ${\beta }_{art}^{^{\prime}}$, which were estimated by the proposed model and the previous model, respectively.

Figure 4A shows the measured and analysed signals from Participant A during the application of electrocutaneous stimulations at frequencies of 5 Hz, 125 Hz, and 250 Hz. We found that blood pressure increased and photoplethysmogram amplitude decreased when the stimuli were applied. The same was observed for the other stimulation durations, which indicated that peripheral arterial stiffness increased when the electrocutaneous stimuli were applied. This interpretation was confirmed from the subfigure (a-2), whereby both estimated peripheral arterial stiffnesses ${\beta }_{art}$ and ${\beta }_{art}^{^{\prime}}$ increased during stimulation. The figure also shows increased stiffness after the stimulation. The increase in stiffness could thus be caused by the rating task, which may be accompanied by recall of the pain stimulus. The wave profiles of ${\beta }_{art}$ and ${\beta }_{art}^{^{\prime}}$ were synchronised and strongly correlated (5 Hz: *r* = 0.93, *p* < 0.001; 125 Hz: *r* = 0.95, *p* < 0.001; 250 Hz: *r* = 0.95, *p* < 0.001). The estimated ${\beta }_{art}$ and ${\beta }_{art}^{^{\prime}}$ for all participants at all stimulation frequencies were strongly correlated (*r* = 0.98, *p* < 0.001; Fig. 4B). This indicates that the proposed model, which can be applied to biological signals measured from fMRI-compatible instruments, can estimate peripheral arterial stiffness with equivalent accuracy to the previous model.

### Experiment 2: Analysis of the relationships between ${\upbeta }_{\text{art}}$, brain activity, self-reported pain intensity, and stimulation amplitude

**Figure 5:**
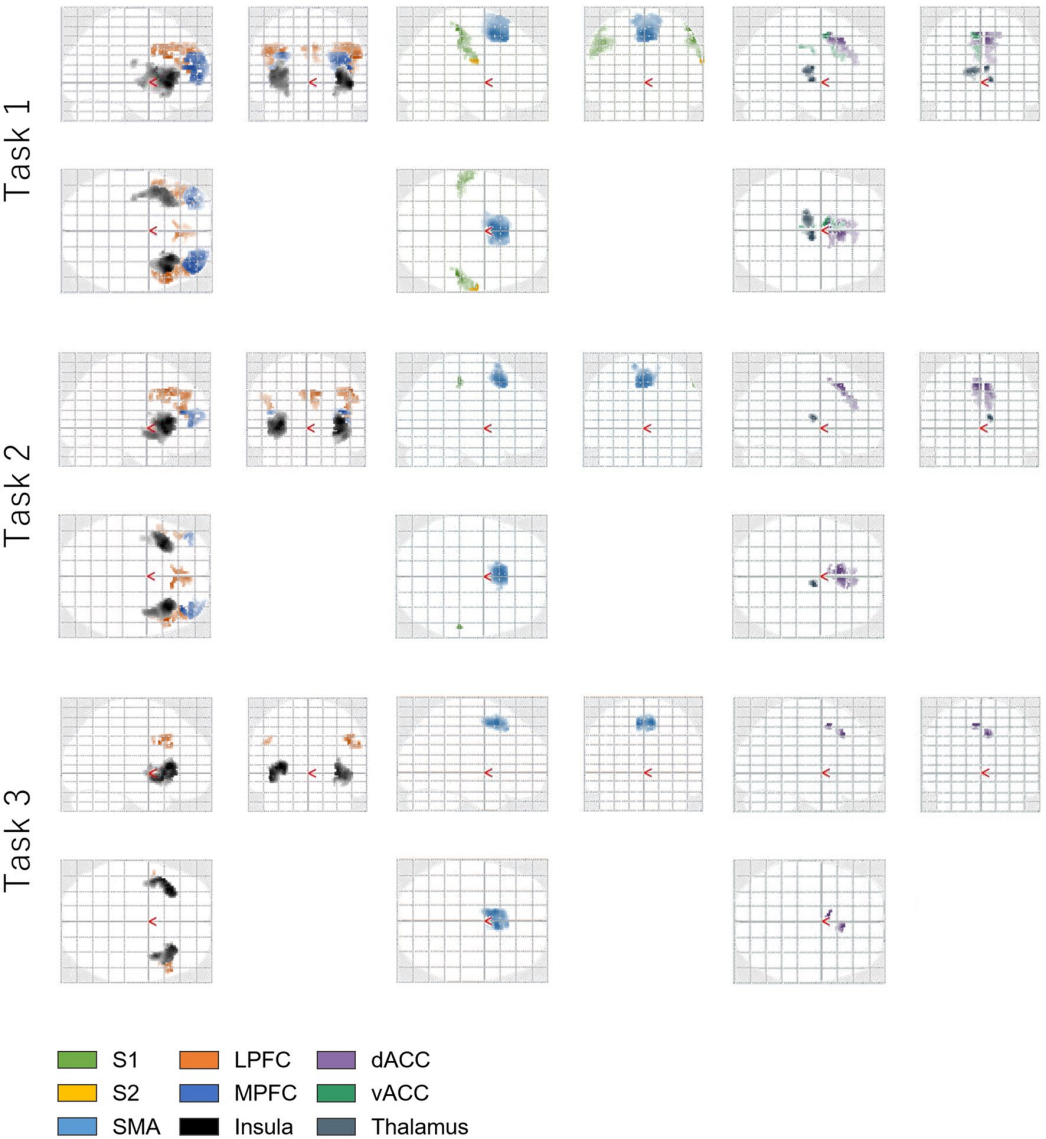

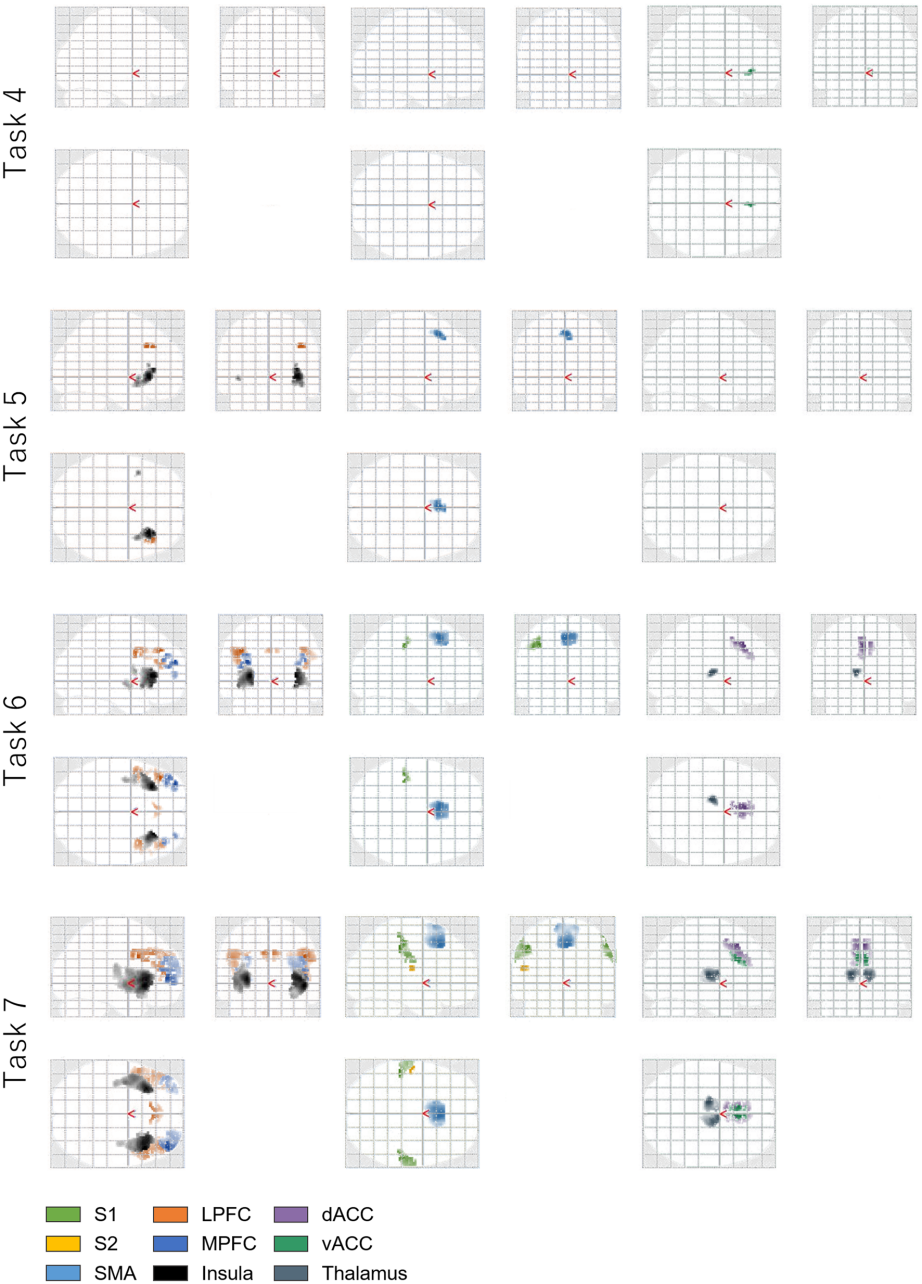
Cerebral activation during electrocutaneous stimulation masked by the pain matrix^6–8^. Activity is shown by the colour graduation in 3-dimensional coordinates of the transparent standard brain. Each subfigure in each task views the brain from the left, superior, and anterior directions. Cerebral activation during electrocutaneous stimulation masked by the pain matrix. Activity is shown by the colour graduation in 3-dimensional coordinates of the transparent standard brain. Each subfigure in each task views the brain from the left, superior, and anterior directions.

Prior to brain activity analysis, we confirmed that head movements were within 20% of the voxel size of 3 × 3 × 4 mm (please refer to Supplemental Information S3). Tables 1, Table 2, Table 3, Table 4, Table 5, Table 6 and Table 7Tables 1–7 show the MNI coordinates of the activated clusters in response to different stimulation amplitudes, whereby the threshold values of uncorrected *p* and cluster extent *k* were set as smaller than 0.001 and more than 20, respectively. Figure 5 shows brain activity masked by the pain matrix (SI, SII, ACC, LPFC, MPFC, insula, SMA, and thalamus) during electrocutaneous stimulation. The whole-brain activity without masking is shown in Supplemental Information S4. Overall, brain activity decreased and the activated area became narrower with decreasing stimulation amplitudes. These results revealed that the activated regions included those that comprise the pain matrix.

**Table 1:** MNI coordinates of the activated clusters in trial 1.

Region	BA	MNI			Peak*Z*	Voxels
**Primary somatosensory Cortices**						
R Inferior parietal gyrus	2	50	− 34	56	7.73	410
L Inferior parietal gyrus	2	− 52	− 30	42	6.32	613
**Secondary somatosensory Cortices**						
R Postcentral gyrus	43	62	− 10	22	5.51	39
L Postcentral gyrus	43	− 52	− 18	18	4.54	35
Lateral prefrontal cortex						
R Inferior frontal gyrus	9	54	20	28	7.46	605
L Precentral gyrus	9	− 46	2	34	7.21	423
R Superior frontal gyrus	9	6	32	38	6.33	86
R Middle frontal gyrus	46	46	40	24	6.16	167
L Middle frontal gyrus	9	− 30	42	30	6.07	24
L Middle frontal gyrus	46	− 42	38	24	5.44	137
**L Superior frontal gyrus**						
Medial prefrontal cortex	9	− 8	26	36	5.24	67
R Superior frontal gyrus		6	32	44	7.13	403
**Insula**						
L Insula	13	− 30	22	8	6.56	1219
R Insula	13	32	22	8	6.55	905
**Supplementary motor area**						
L Supplementary motor area	32	− 4	6	52	7.74	2799
**Thalamus**						
L Thalamus		− 12	− 16	8	5.20	482
R Thalamus		10	− 14	0	4.99	274

In this trial, a stimulation current amplitude that was 1.5 times that of the standard amplitude was applied. Threshold values of uncorrected *p* and cluster extent *k* were set as smaller than 0.001 and more than 20, respectively.

*L* left hemisphere; *R* right hemisphere.

**Table 2:** MNI coordinates of the activated clusters in trial 2.

Region	BA	MNI			Peak *Z*	Voxels
		*x* [mm]	*y* [mm]	*z* [mm]		
**Primary somatosensory cortices**						
R Supramarginal gyrus	2	58	− 30	50	4.23	40
**Lateral prefrontal cortex**						
R Inferior frontal gyrus	9	50	20	38	6.01	325
R Cingulate gyrus	9	10	26	36	5.47	132
R Middle frontal gyrus	46	38	36	16	5.17	106
L Superior frontal gyrus	9	− 2	32	38	4.97	68
R Inferior frontal gyrus	9	44	10	32	4.91	78
L Middle frontal gyrus	46	− 42	38	22	4.69	70
L Inferior frontal gyrus	9	− 48	16	26	4.35	141
L Middle frontal gyrus	9	− 36	20	34	3.93	22
**Medial prefrontal cortex**						
R Superior frontal gyrus	6	4	32	42	5.44	413
**Insula**						
R Insula	13	36	22	6	6.75	876
L Insula		− 34	16	2	6.67	842
L Insula	13	− 40	0	− 6	3.86	66
**Supplementary motor area**						
L Supplementary motor area		− 2	16	48	6.42	1050
**Thalamus**						
R Thalamus		10	− 10	10	4.10	128
L Thalamus		− 10	− 18	12	3.82	36

In this trial, a stimulation current amplitude that was 1.0 times that of the standard amplitude was applied. Threshold values of uncorrected *p* and cluster extent *k* were set as smaller than 0.001 and more than 20, respectively.

*L* left hemisphere; *R* right hemisphere.

**Table 3:** MNI coordinates of the activated clusters in trial 3.

Region	BA	MNI			Peak *Z*	Voxels
		*x* [mm]	*y* [mm]	*z* [mm]		
**Lateral prefrontal cortex**						
R Inferior frontal gyrus	9	44	10	32	5.05	83
R Inferior frontal gyrus	9	44	16	36	4.57	87
L Precentral gyrus	9	− 48	4	32	4.20	43
**Medial prefrontal cortex**						
R Superior frontal gyrus	8	4	26	48	4.14	103
**Insula**						
L Insula	45	− 32	24	6	5.09	684
R Insula		32	26	0	4.81	688
**Supplementary motor area**						
R Supplementary motor area		8	14	52	5.69	1128

In this trial, a stimulation current amplitude that was 0.5 times that of the standard amplitude was applied. Threshold values of uncorrected *p* and cluster extent *k* were set as smaller than 0.001 and more than 20, respectively.

*L* left hemisphere; *R* right hemisphere.

**Table 4:** MNI coordinates of the activated clusters in trial 4.

Region	BA	MNI			Peak *Z*	Voxels
		*x* [mm]	*y* [mm]	*z* [mm]		
L Inferior occipital gyrus		− 24	− 90	− 10	5.92	731
R Anterior cingulum gyrus	24	4	32	− 2	4.07	123
R Calcarine gyrus		20	− 92	0	4.03	407
L Precentral gyrus		− 32	− 22	60	4.02	191
L Middle temporal gyrus	22	− 56	− 32	2	3.64	32

In this trial, a stimulation current amplitude that was 0 times that of the standard amplitude was applied. Threshold values of uncorrected *p* and cluster extent *k* were set as smaller than 0.001 and more than 20, respectively.

*L* left hemisphere; *R* right hemisphere.

**Table 5:** MNI coordinates of the activated clusters in trial 5.

Region	BA	MNI			Peak *Z*	Voxels
		*x* [mm]	*y* [mm]	*z* [mm]		
**Primary somatosensory cortices**						
L Postcentral gyrus	2	− 38	− 30	44	4.31	184
**Lateral prefrontal cortex**						
L Middle frontal gyrus	9	− 40	36	36	5.45	49
L Middle frontal gyrus	9	− 34	44	32	4.78	21
L Precentral gyrus	9	− 46	4	34	4.76	152
R Inferior frontal gyrus	46	44	38	24	4.64	85
L Inferior frontal gyrus	46	− 46	32	24	4.43	52
R Middle frontal gyrus	9	40	4	38	4.29	211
L Superior frontal gyrus	9	− 4	30	38	4.27	32
R Middle frontal gyrus	9	38	38	36	4.23	70
R Superior frontal gyrus	9	4	32	38	3.70	30
**Medial prefrontal cortex**						
L Superior frontal gyrus	8	− 4	30	44	4.65	255
**Insula**						
R Insula		30	28	4	5.79	465
L Insula	45	− 30	24	6	5.74	760
L Insula		− 38	− 6	− 8	4.85	142
R Insula		40	0	− 6	3.48	92
**Supplementary motor area**						
L Supplementary motor area		− 6	14	50	5.30	939
**Thalamus**						
L Thalamus		− 12	− 16	8	4.61	153
R Thalamus		12	− 12	10	3.77	63

In this trial, a stimulation current amplitude that was 0.5 times that of the standard amplitude was applied. Threshold values of uncorrected *p* and cluster extent *k* were set as smaller than 0.001 and more than 20, respectively.

*L* left hemisphere; *R* right hemisphere.

**Table 6:** MNI coordinates of the activated clusters in trial 6.

Region	BA	MNI			Peak *Z*	Voxels
		x [mm]	y [mm]	z [mm]		
**Primary somatosensory cortices**						
R Supramarginal gyrus	2	60	− 32	40	3.60	23
**Lateral prefrontal cortex**						
R Middle frontal gyrus	9	40	22	36	4.15	160
R Middle frontal gyrus	46	44	38	22	4.12	21
L Inferior frontal gyrus	9	− 46	4	28	4.10	27
L Middle frontal gyrus	46	− 46	40	22	3.80	31
R Inferior frontal gyrus	46	46	16	26	3.72	20
**Medial prefrontal cortex**						
R Medial Superior frontal gyrus	8	4	28	46	4.60	111
**Insula**						
R Insula		34	22	0	4.56	397
L Insula	13	− 42	10	− 4	4.02	316
**Supplementary motor area**						
L Supplementary motor area		0	14	52	4.72	616
**Thalamus**						
L Thalamus		− 18	− 18	10	4.44	58

In this trial, a stimulation current amplitude that was 1.0 times that of the standard amplitude was applied. Threshold values of uncorrected *p* and cluster extent *k* were set as smaller than 0.001 and more than 20, respectively.

*L* left hemisphere; *R* right hemisphere.

**Table 7:** MNI coordinates of the activated clusters in trial 7.

Region	BA	MNI			Peak *Z*	Voxels
		*x* [mm]	*y* [mm]	*z* [mm]		
**Primary somatosensory cortices**						
L Inferior parietal gyrus	2	− 48	− 32	42	5.98	186
R Supramarginal gyrus	2	58	− 28	48	5.94	229
**Secondary somatosensory cortices**						
L Postcentral gyrus	43	− 56	− 14	16	4.28	36
**Lateral prefrontal cortex**						
R Middle frontal gyrus	46	46	48	6	6.63	133
R Inferior frontal gyrus	9	44	10	30	6.15	389
L Superior frontal gyrus	9	− 8	26	36	5.55	54
R Cingulate gyrus	9	4	30	38	5.42	57
L Precentral gyrus	9	− 48	10	30	5.26	187
L Inferior frontal gyrus	46	− 46	32	24	4.86	121
L Middle frontal gyrus	9	− 30	52	32	4.82	20
L Middle frontal gyrus	9	− 42	24	34	4.71	89
**Medial prefrontal cortex**						
R Superior frontal gyrus	8	4	26	48	7.10	1577
**Insula**						
R Insula		46	20	− 4	6.77	1193
L Insula		− 30	22	2	6.57	1383
L Insula		− 28	8	− 16	3.70	20
**Supplementary motor area**						
R Supplementary motor area		2	20	48	7.10	1577
**Thalamus**						
L Thalamus		− 12	− 16	8	5.22	320
R Thalamus		8	− 14	10	5.10	351

In this trial, a stimulation current amplitude that was 1.5 times that of the standard amplitude was applied. Threshold values of uncorrected *p* and cluster extent *k* were set as smaller than 0.001 and more than 20, respectively.

*L* left hemisphere; *R* right hemisphere.

**Figure 6:**
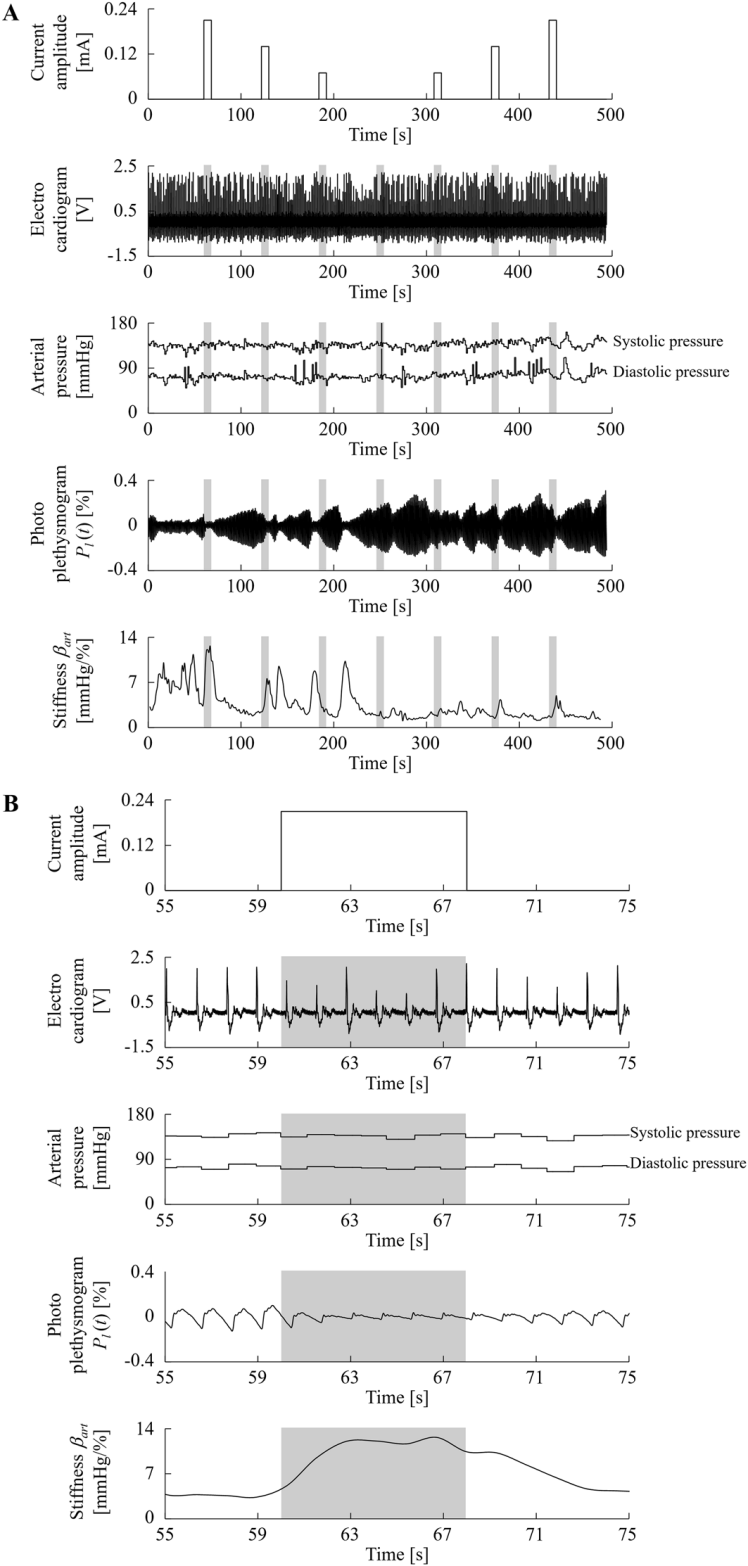
Electrocardiograms, systolic and diastolic blood pressure, photo-plethysmography, and estimated stiffness (*β*_art_) of Participant A in session 3. (**A**) The entire duration of session 3; (**B**) 55 s to 75 s, corresponding to the first electrocutaneous stimulation task with the strongest electrocutaneous stimulus (1.5 × stimulation).

Figure 6 shows the maximum current amplitude of the electrocutaneous stimulation and measured biological signals (systolic blood pressure *P*_SYS_, diastolic blood pressure *P*_dia_, and photo-plethysmography), and estimated ${\beta }_{art}$ obtained from Participant A. We confirmed that the blood volume measured by photo-plethysmography decreased and ${\beta }_{\text{art}}$ increased by 10 mmHg/% until reaching the maximum when the strongest stimulus was applied. The same was observed for the other non-zero stimulation amplitudes, which indicates that peripheral arterial stiffness increased during electrocutaneous stimulation.

**Figure 7:**
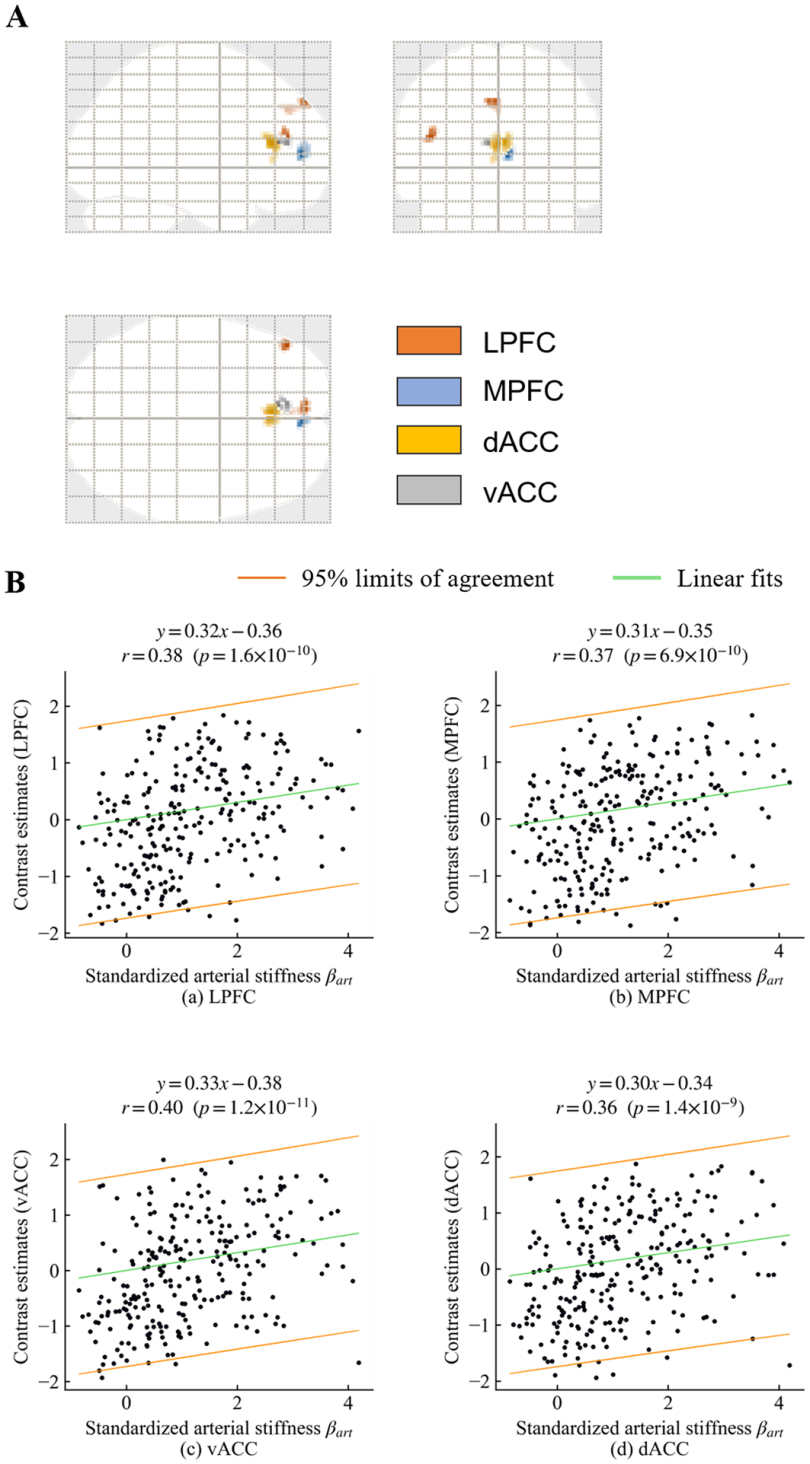
Parametric analysis results. (**A**) The brain regions in which BOLD signals covaried with peripheral arterial stiffness. The regions that are consistent with the pain matrix are highlighted by warm colours. (**B**) The relationship between standardised peripheral arterial stiffness (*β*_art_) and average contrast estimates in the significant clusters of voxels that were consistent with the pain matrix.

Figure 7 shows the brain regions in which the BOLD signal covaried with the peripheral arterial stiffness ${\beta }_{\text{art}}$ obtained by the parametric modulation analysis. Significant clusters of voxels that were consistent with pain matrix were found in the LPFC, MPFC, dorsal ACC (dACC), and ventral ACC (vACC), as shown in Fig. 7A. Figure 7B shows scatter plots depicting the average contrast estimates and the maximum ${\beta }_{\text{art}}$ for each stimulus duration. We found low to moderate correlations (LPFC: $r=0.38, p=1.6\times {10}^{-10}$; MPFC: $r=0.37, p=6.9\times {10}^{-10}$; vACC: $r=0.40, p=1.2\times {10}^{-11}$; dACC: $r=0.36, p=1.4\times {10}^{-9}$). These results revealed the brain regions associated with both pain perception and peripheral arterial stiffness.

**Figure 8:**
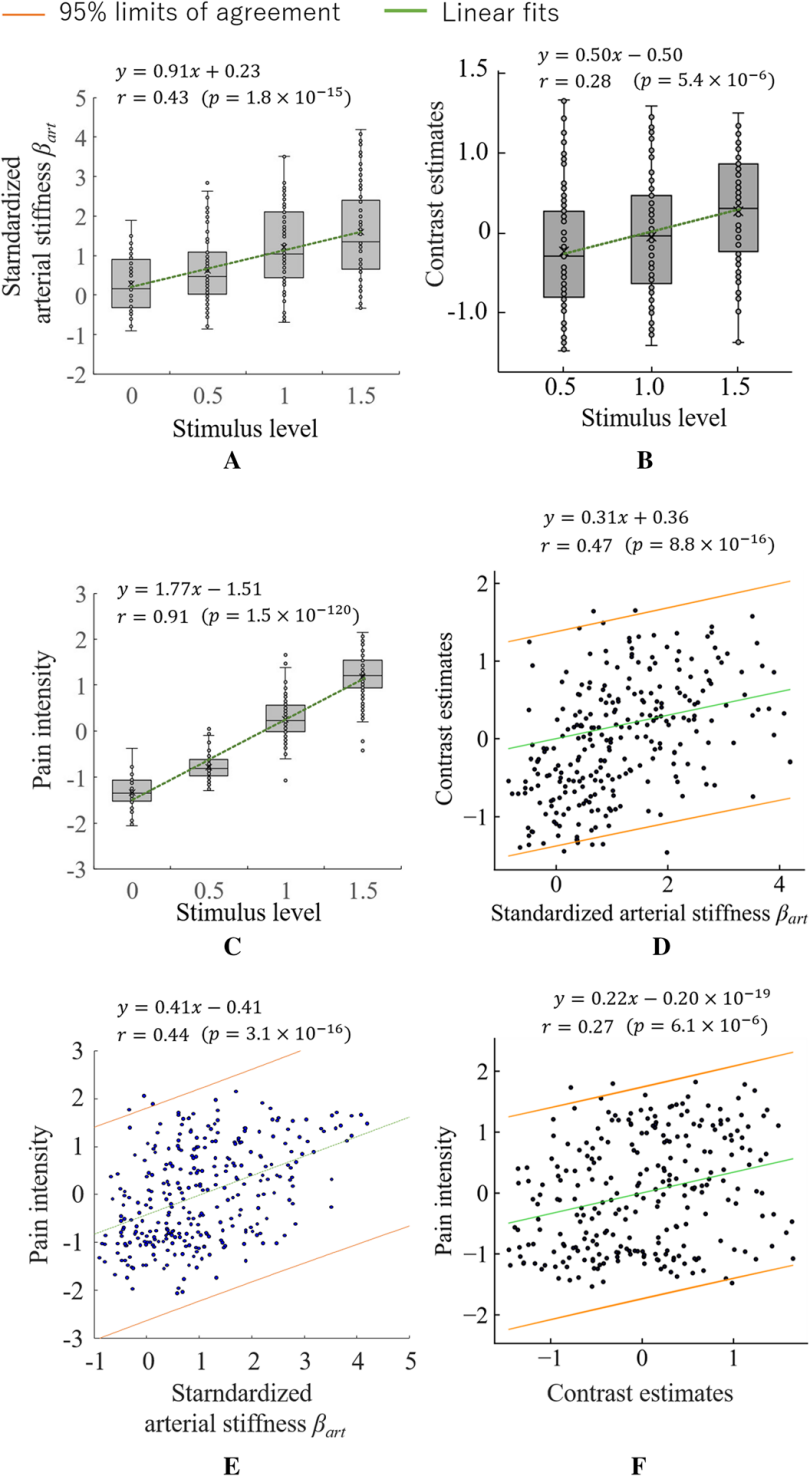
Correlations between standardised stimulation level, peripheral arterial stiffness parameter (*β*_art_), contrast estimates averaged over all pain matrix-related areas, and self-reported pain intensity (VAS scores).

Figure 8 shows the comparison between each pair of the average contrast estimates, *β*_art_, stimulation amplitude, and self-reported pain intensity. The contrast estimates were averaged over the four significant clusters of voxels that were consistent with the pain matrix. Each pair of variables showed a low to moderate correlation (stimulus level and *β*_art_: *r* = 0.43, *p* < 0.001; stimulus level and contrast estimates: *r* = 0.28, *p* < 0.001; stimulus level and pain intensity: *r* = 0.91, *p* < 0.001; *β*_art_ and contrast estimates: *r* = 0.47, *p* < 0.001; *β*_art_ and pain intensity: *r* = 0.44, *p* < 0.001; contrast estimates and pain intensity: *r* = 0.46, *p* < 0.001).

## Discussion

To explore the neurological and psychological correlations between peripheral arterial stiffness and pain stimulation, we proposed a model (Eq. (2)) to estimate peripheral arterial stiffness ${\beta }_{art}$, which reflects peripheral sympathetic nervous activity. This model is a simplified version of our previous model^11^ that allows peripheral arterial stiffness to be estimated using biological signals that can be measured in an fMRI environment. A correlation analysis was then performed between ${\beta }_{art}$, pain-related brain activity, self-reported pain intensity, and stimulation level.

First, we verified the accuracy of the proposed model for estimating the peripheral arterial stiffness. There was a strong correlation between ${\beta }_{art}$ estimated by the proposed model (Eq. (2)) and ${\beta }_{art}^{^{\prime}}$ estimated by the previous model (Eq. (2)) (see Fig. 4A). Although some noise, possibly caused by body movement, was observed in the *P*_SYS_ and *P*_dia_ measurements (see Fig. 6), the calculated ${\beta }_{art}$ was not greatly influenced by this. We thus confirmed that the model can be used for simultaneous fMRI acquisition and ${\beta }_{art}$ estimation.

We next performed experiments in the fMRI environment. The contrast estimates between stimulus and rest duration showed that a higher stimulus level resulted in stronger and more widespread brain responses, and that the pain matrix was among the regions that responded (see Fig. 5 and Tables 1–7). Parametric analysis showed that four brain regions (the LPFC, MPFC, vACC, and dACC) with BOLD signals that covaried with ${\beta }_{\text{art}}$ were consistent with the pain matrix (see Fig. 7). Moderate correlations were observed between ${\beta }_{\text{art}}$ and the contrast estimates in these regions. These results indicate that ${\beta }_{art}$ is positively correlated with brain activity evoked by painful electrocutaneous stimuli.

There was a strong correlation between stimulus level and self-reported pain intensity; however, other correlations between each pair of the average contrast estimates, *β*_art_, stimulation amplitude, and self-reported pain intensity, were moderate (see Fig. 8). This could be partially caused by individual differences in nociception^20^.

Both Experiment 1 and Experiment 2 showed that stimulation decreased the blood volume measured by photo-plethysmography and increased ${\beta }_{art}$, which is consistent with our previous reports that peripheral arterial walls contract in response to sympathetic nerve activity^21^. However, relationships of ${\beta }_{art}$ with information processing in pain recognition pathways, such as spinothalamic tracts from the skin to the SI and SII via the outer thalamus^8^, are poorly understood. Mulder’s short-term blood pressure regulation model^22^ may provide us with a basis from which to investigate the physiological process of pain perception. This model indicates that the modulation pathway of peripheral arterial stiffness is included in a large closed loop of blood pressure regulation, and is affected by both sympathetic tone and higher brain centres. Thus, the response of peripheral arterial stiffness could be caused by reflexes of sympathetic nerves and/or commands from higher brain centres; applying a causality analysis would allow us to clarify this. However, we have not yet determined which method would be appropriate for such a causality analysis. Given that our experimental configuration included the main physiological measures (brain activity, heart rate, respiration, and peripheral arterial stiffness) used in Mulder’s model, acquiring fMRI data with a higher time resolution should allow us to clarify the physiological causalities.

In addition, peripheral arterial stiffness does not always increase immediately after a pain stimulus, and may not respond to a very short stimulus. Therefore, stimulus duration may have affected the presented results. In this paper, we chose stimulus durations of 24 and 8 s according to our previous study^12^ and preliminary experiment, respectively. Although we confirmed that peripheral arterial stiffness responds to painful stimuli at a determined stimulation duration, it will be necessary to examine the optimal duration to make use of peripheral arterial stiffness as a predictor of pain. Furthermore, the effects of learning, habituation, and presented context should be investigated in the future. The effects of anxiety on peripheral arterial stiffness and its reliability as a predictor of pain qualities are also open questions.

In this study, participants were restricted to young men. Considering that our previous study indicated that ageing affects the mechanical characteristics of arteries^23^, the lack of analysis on the age and sex could be limitations of this paper.

The present study found weak to strong, but significant, correlations between the stimulation level, peripheral arterial stiffness ${\beta }_{art} ($ which is governed by sympathetic nerve activity), the contrast estimates representing brain activity, and self-reported pain intensity. The correlation of ${\beta }_{art}$ with the contrast estimates and the self-reported pain intensity may help to establish an objective pain evaluation metric.

## Supplementary Information

Supplementary Information.

## Data Availability

The datasets generated during and/or analysed during the current study are available from the corresponding author on reasonable request.
