# Supplementary material for: Peripheral arterial stiffness during electrocutaneous stimulation is positively correlated with pain-related brain activity and subjective pain intensity: an fMRI study
Source: Sci Rep. 2021 Feb 24;11:4425. doi: 10.1038/s41598-021-83833-6 (PMC7904817; doi:10.1038/s41598-021-83833-6)
Supplement: Supplementary file 1 — Supplementary Information. [file 41598_2021_83833_MOESM1_ESM.docx]

**Supplemental Information**

**Manuscript title:**

Peripheral Arterial Stiffness During Electrocutaneous Stimulation is Positively Correlated with Pain-related Brain Activity and Subjective Pain Intensity: An fMRI Study

# **Authors**:

Toshio Tsuji^1*^, Fumiya Arikuni^1^, Takafumi Sasaoka^2^, Shin Suyama^1^, Takashi Akiyoshi^1^, Zu Soh^1^, Harutoyo Hirano^3^, Ryuji Nakamura^4^, Noboru Saeki^4^, Masashi Kawamoto^4^, Masao Yoshizumi^5^, Atsuo Yoshino^6^, and Shigeto Yamawaki^2^

# **Affiliations**:

^1^Department of System Cybernetics, Graduate School of Engineering, Hiroshima University, 1-4-1 Higashi-Hiroshima, Hiroshima 739-8527, Japan

^2^Brain, Mind, and KANSEI Sciences Research Center, Hiroshima University, 1-2-3 Kasumi, Minami-ku, Hiroshima 734-8551, Japan

^3^College of Engineering, Academic Institute, Shizuoka University, 836 Ohtani, Suruga-ku, Shizuoka 422-8529, Japan

^4^Department of Anesthesiology and Critical Care, Graduate School of Biomedical and Health Sciences, Hiroshima University, 1-2-3 Kasumi, Minami-ku, Hiroshima 734-8551, Japan

^5^Department of Cardiovascular Physiology and Medicine, Graduate School of Biomedical and Health Sciences, Hiroshima University, 1-2-3 Kasumi, Minami-ku, Hiroshima 734-8553, Japan

^6^Department of Psychiatry and Neurosciences, Graduate School of Biomedical and Health Sciences, Hiroshima University, 1-2-3 Kasumi, Minami-ku, Hiroshima 734-8551, Japan

*Corresponding author: Toshio Tsuji and Zu Soh

Address: Department of System Cybernetics, Graduate School of Engineering, Hiroshima University, 1-4-1 Higashi-Hiroshima, Hiroshima 739-8527, Japan

E-mail: tsuji@bsys.hiroshima-u.ac.jp and sozu@bsys.hiroshima-u.ac.jp

Phone: +81-82-424-7676

Fax: +81-82-424-2387

## **S1: Approximation process to derive peripheral arterial stiffness estimation model (Eq. (2))**

The log-linearised peripheral arterial viscoelastic model is defined by the following equation [Matsubara et al. *Sci Rep*, 2018]:

$$P_{b}\left( t \right)=\mu\ddot{P}_{l}\left( t \right)+\eta\dot{P_{l}}\left( t \right)+\exp\left\{ \beta_{art}^{'}P_{l}\left( t \right)+P_{b\beta_{art0}^{'}}+P_{b\beta_{artnl}^{'}}\left( P_{l}\left( t \right) \right) \right\}, (1)$$

where $P_{b}\left( t \right)$ is the continuous arterial pressure, $P_{l}\left( t \right)$ is the photoplethysmogram, $P_{b\beta_{art0}^{'}}$ is the constant pressure component, $P_{b\beta_{artnl}^{'}}\left( P_{l}\left( t \right) \right)$ is the nonlinear stiffness pressure component originating in the vein, $\mu$ is the inertia, $\eta$ is the viscosity, and $\beta_{art}^{'}$ is the peripheral arterial stiffness. In addition, *t* represents time and the dot operator on $P_{l}\left( t \right)$ represents the time derivative.

Let us consider the case where stiffness is dominant in comparison with viscosity and inertia. When the arterial pressure is at its maximum ($P_{b}\left( t \right)=P_{sys}$) and minimum ($P_{b}\left( t \right)=P_{dia}$), we approximate $P_{l}\left( t \right)=P_{l max} \mathrm{and} {P_{l}\left( t \right)=P}_{l min}$, respectively, and $\dot{P_{l}}\left( t \right)=0$. Provided that the inertia of the arterial wall is very small and negligible, the following two equations were then derived from Eq. (1):

$$P_{sys}=\exp\left\{ \beta_{art}^{'}P_{l max}+P_{b\beta_{art0}^{'}}+P_{b\beta_{artnl}^{'}}\left( P_{l max} \right) \right\},$$

$$P_{dia}=\exp\left\{ \beta_{art}^{'}P_{l min}+P_{b\beta_{art0}^{'}}+P_{b\beta_{artnl}^{'}}\left( P_{l min} \right) \right\}.$$

Taking the natural logarithm of both sides gives

$${ln(P}_{sys})=\beta_{art}^{'}P_{l max}+P_{b\beta_{art0}^{'}}+P_{b\beta_{artnl}^{'}}\left( P_{l max} \right), (a)$$

$${ln(P}_{dia})=\beta_{art}^{'}P_{l min}+P_{b\beta_{art0}^{'}}+P_{b\beta_{artnl}^{'}}\left( P_{l min} \right). (b)$$

When we assume $P_{b\beta_{artnl}^{'}}\left( P_{l max} \right)$ ≈ $P_{b\beta_{artnl}^{'}}\left( P_{l min} \right)$, subtracting (b) from (a) gives

$${ln(P}_{sys})-{ln(P}_{dia})=\beta_{\mathrm{art}}(P_{l max}-P_{l min}),$$

where $\beta_{\mathrm{art}}$ represents the approximated $\beta_{art}^{'}$. Peripheral arterial stiffness can thus be estimated using the following equation:

$$\beta_{\mathrm{art}}=\frac{\ln\left( \frac{P_{sys}}{P_{dia}} \right)}{P_{l max}-P_{l min}} (2)$$

The parameters were extracted from the measured biological signals (photo-plethysmogram and blood pressure) on a beat-to-beat basis, as shown in the following figure. ECG was used to obtain the R-R interval.

|   Fig. S1-1 Segmentation of measured biosignals based on R-R intervals to estimate peripheral arterial -stiffness $\beta_{\mathrm{art}}$.  The figure shows electrocardiogram (ECG), photo-plethysmogram, and radial artery pressure in descending order. Systolic blood pressure ($P_{\mathrm{SYS}}$), diastolic blood pressure ($P_{\mathrm{dia}}$), maximum amplitude of photo-plethysmogram ($P_{\mathrm{lmax}}$), and minimum amplitude of photo-plethysmogram ($P_{\mathrm{lmin}}$) were extracted from each R-R interval. |
| --- |

## **S2: Supplementary method: fMRI data analysis**

Image processing and statistical analysis were performed using SPM12 software (Wellcome Department of Cognitive Neurology, www.fil.ion.ucl.ac.uk/spm) running on MATLAB R2015b (Mathworks, Natick, MA). We discarded the first 5 volumes of functional images to allow for T1 equilibration. We first spatially realigned to the first volume to correct for head movements. We then corrected slice-timing to allow the analysis to proceed as though the whole brain had been imaged simultaneously. T1-weighted anatomical images were co-registered to the first functional images. Co-registered anatomical images were spatially normalised to the Montreal Neurological Institute (MNI) template using the registered structural images to match the standard brain. Parameters derived from this normalisation process were then applied to each functional image. At the end of pre-processing, the normalised functional images were spatially smoothed using a Gaussian kernel with a full width at half maximum of 8 mm. This corrected individual brain differences that could not be corrected using standardisation and reduced noise in the fMRI data without compromising spatial resolution.

Pre-processed echo-planar images [17] were analysed using a general linear model. Each period of electrocutaneous stimulus and rest was modelled with a boxcar function, and linear predictors were created with a convolution of the haemodynamic response function. Prior to the regression analysis, the low-frequency BOLD signal fluctuation was removed by applying a high-pass filter with a cut-off of 128 seconds. The serial correlation between scans was also removed using a first-order autoregressive model.

## **S3: Head motion**

It should be noted that 6 parameters related to head movement, obtained by the realignment process, were included in the model as linear predictors to remove head movement-related artifacts. The following figure shows an example of the time plot of the head motion of the participant with the largest displacement. The head movement was within 20% of the voxel size of 3 × 3 × 4 mm.

|   Fig. S3-1 Head motion of the participant with the largest displacement. The upper plot shows the translational displacement where the blue, green, and red lines denote the *x*, *y*, and *z* axes, respectively. The lower plot shows the rotational displacement where the blue, green, and red lines denote pitch, roll, and yaw axes, respectively. |
| --- |

# **S4: Results of the region of interest analysis**

The regions of interest (ROIs) were determined on the basis of the anatomical locations of the SI, SII, LPFC, MPFC, insula, supplementary motor area (SMA), and thalamus using MNI coordinates. The contrast estimates were calculated for the voxels within a 3 mm radius centred on the peak coordinates specified in the individual-level analysis of the first trial. This analysis was carried out using MarsBaR SPM toolbox (<http://marsbar.sourceforge.net/>) [16].

Tables 1–7 in the main text show the MNI coordinates of the activated clusters in response to different stimulation amplitudes, where the threshold values of uncorrected *p* and cluster extent *k* were set as smaller than 0.001 and more than 20, respectively. Figure S4-1 shows brain activity during electrocutaneous stimulation. Overall, brain activity decreased and the activated area became narrower with decreasing stimulation amplitudes. When the strongest (1.5× stimulation) was applied in trials 1 and 7, all ROIs were significantly activated. When the moderate stimulation (1.0× stimulation) was applied, the activated ROIs differed between trial 2 and trial 6. That is, in trial 6, significant activations were confirmed in all ROIs; however, in trial 2, significant activation was not confirmed for the SII. These asymmetric activations between the earlier and the later conditions were also observed when the weakest stimulation (0.5× stimulation) was applied; in trial 3, all ROIs except for the SI, SII, and thalamus exhibited significant activation, and in trial 5, all ROIs except for the SII exhibited significant activation. When no stimulation was applied (0× stimulation in trial 4), the inferior occipital, anterior cingulate, calcarine, precentral, and middle temporal gyri were significantly activated, but no significant activation was found in the other ROIs. This suggests that there was an order effect of the applied electrocutaneous stimuli.

The contrast estimates in each ROI were correlated with $\beta_{art}$ (SI: *r* = 0.34, *p* < 0.001; SII: *r* = 0.35, *p* < 0.001; LPFC: *r* = 0.45, *p* < 0.001; MPFC: *r* = 0.43, *p* < 0.001; insula: *r* = 0.44, *p* < 0.001; SMA: *r* = 0.42, *p* < 0.001; thalamus: *r* = 0.41, *p* < 0.001; Fig. S4-2). Additionally, contrast estimates averaged over all ROIs increased the correlation coefficient (*r* = 0.55, *p* < 0.001; Fig. S4-1 (h)). Figure S4-2 shows the comparison between each pair of the average contrast estimates, *β*_art_, stimulation amplitude, and self-reported pain intensity. Each pair of variables showed a moderate partial correlation (stimulation amplitude and *β*_art_: *r* = 0.43, *p* < 0.001; stimulation amplitude and contrast estimates: *r* = 0.48, *p* < 0.001; stimulation amplitude and pain intensity: *r* = 0.91, *p* < 0.001; *β*_art_ and contrast estimates: *r* = 0.55, *p* < 0.001; *β*_art_ and pain intensity: *r* = 0.44, *p* < 0.001; contrast estimates and pain intensity: *r* = 0.46, *p* < 0.001). These results indicate that the peripheral arterial stiffness estimated by the approximated model was neurologically and psychologically correlated with self-reported pain.

These results also support the finding that peripheral arterial stiffness was positively correlated with pain-related brain activity and subjective pain intensity, as reported in the main text.


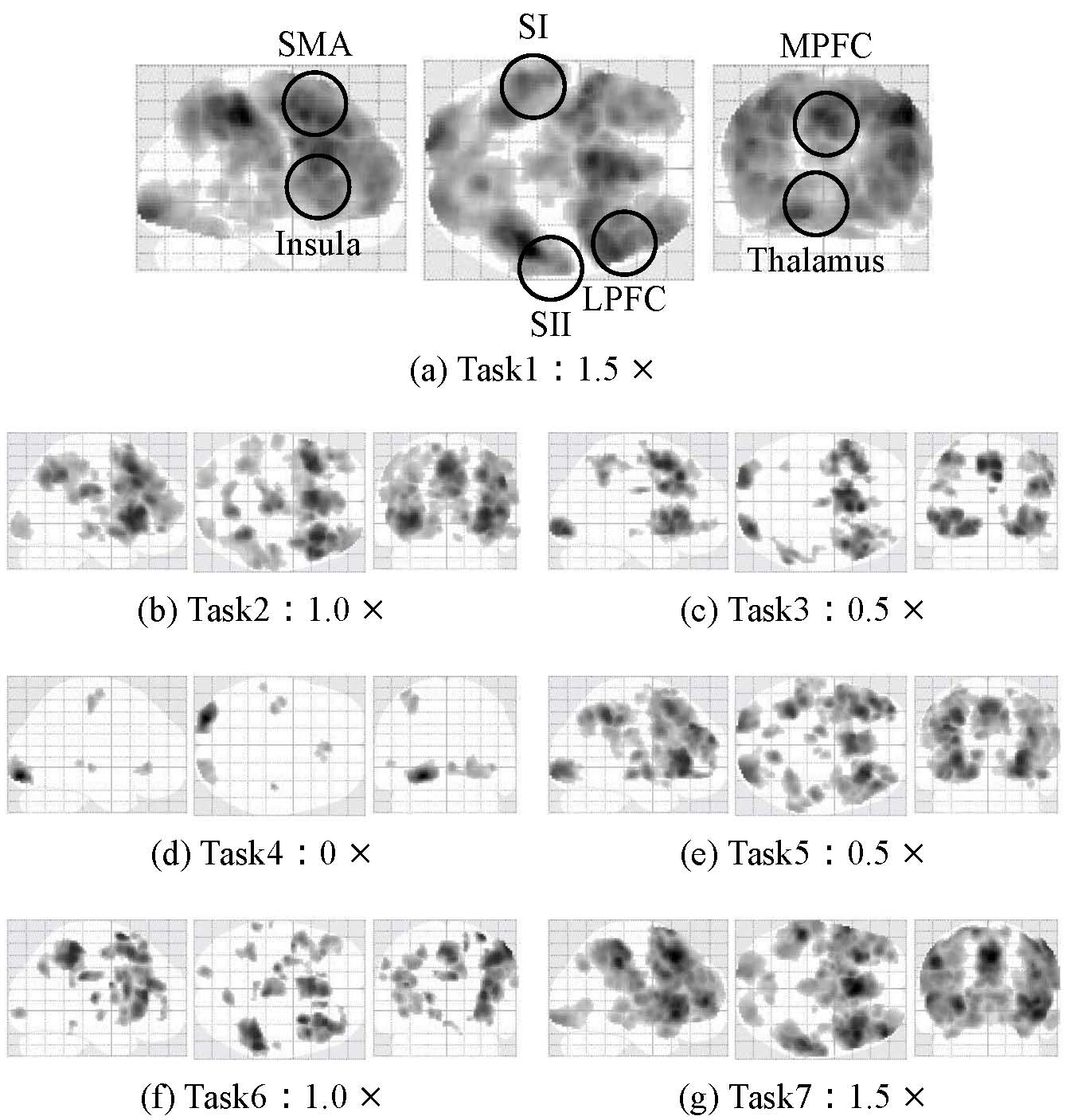


Figure S4-1. Cerebral activation by electrocutaneous stimuli. Activity is shown by the grey scale in 3-dimensional coordinates of the transparent standard brain. Each subfigure in each task views the brain from the left, superior, and anterior directions.


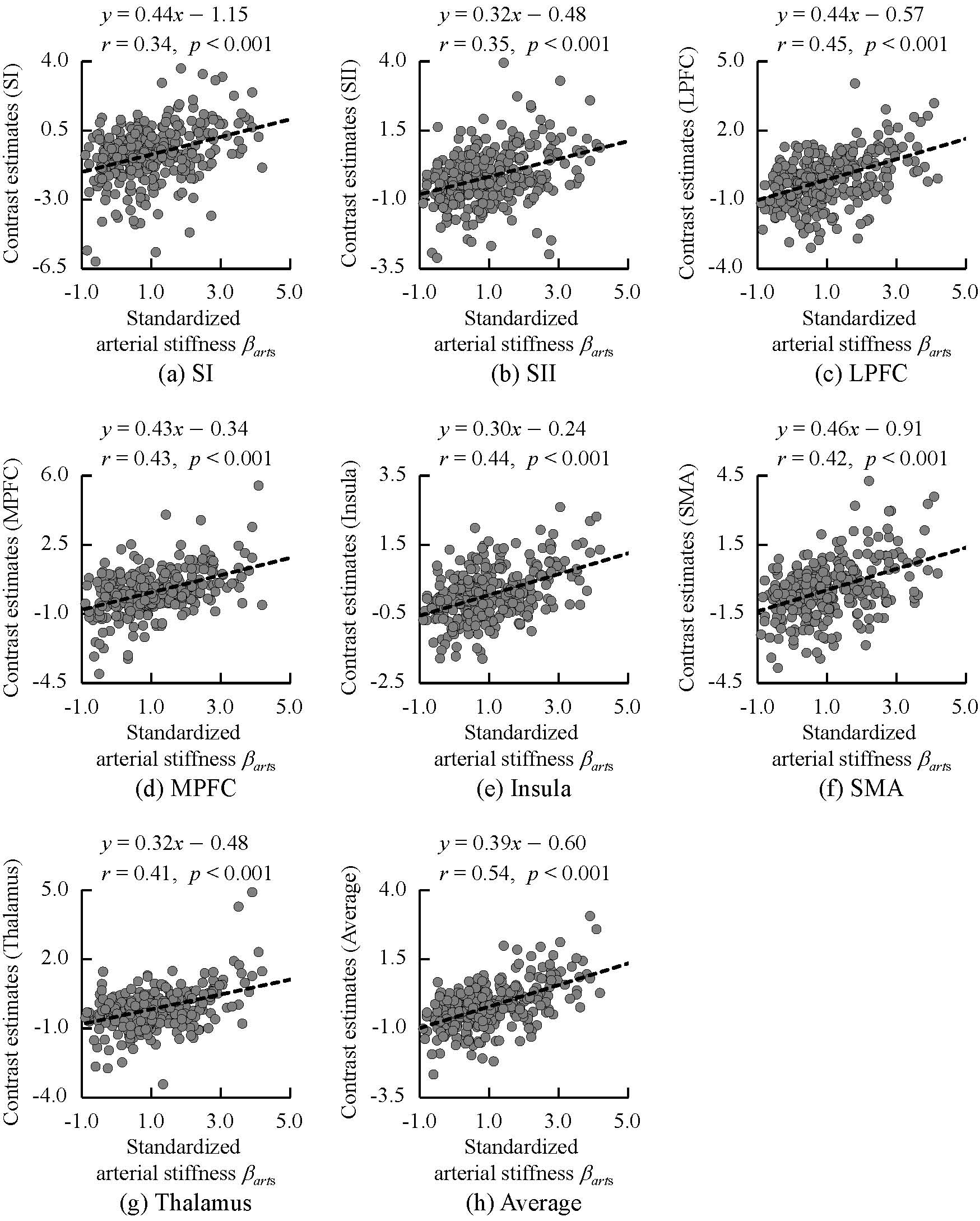


Figure S4-2. The relationship between standardised peripheral arterial stiffness (*β*_art_) and contrast estimates in the primary (SI) and secondary (SII) somatosensory cortices, lateral prefrontal cortex (LPFC), medial prefrontal cortex (MPFC), insula, supplementary motor area (SMA), and thalamus, and average contrast estimates for all ROIs.


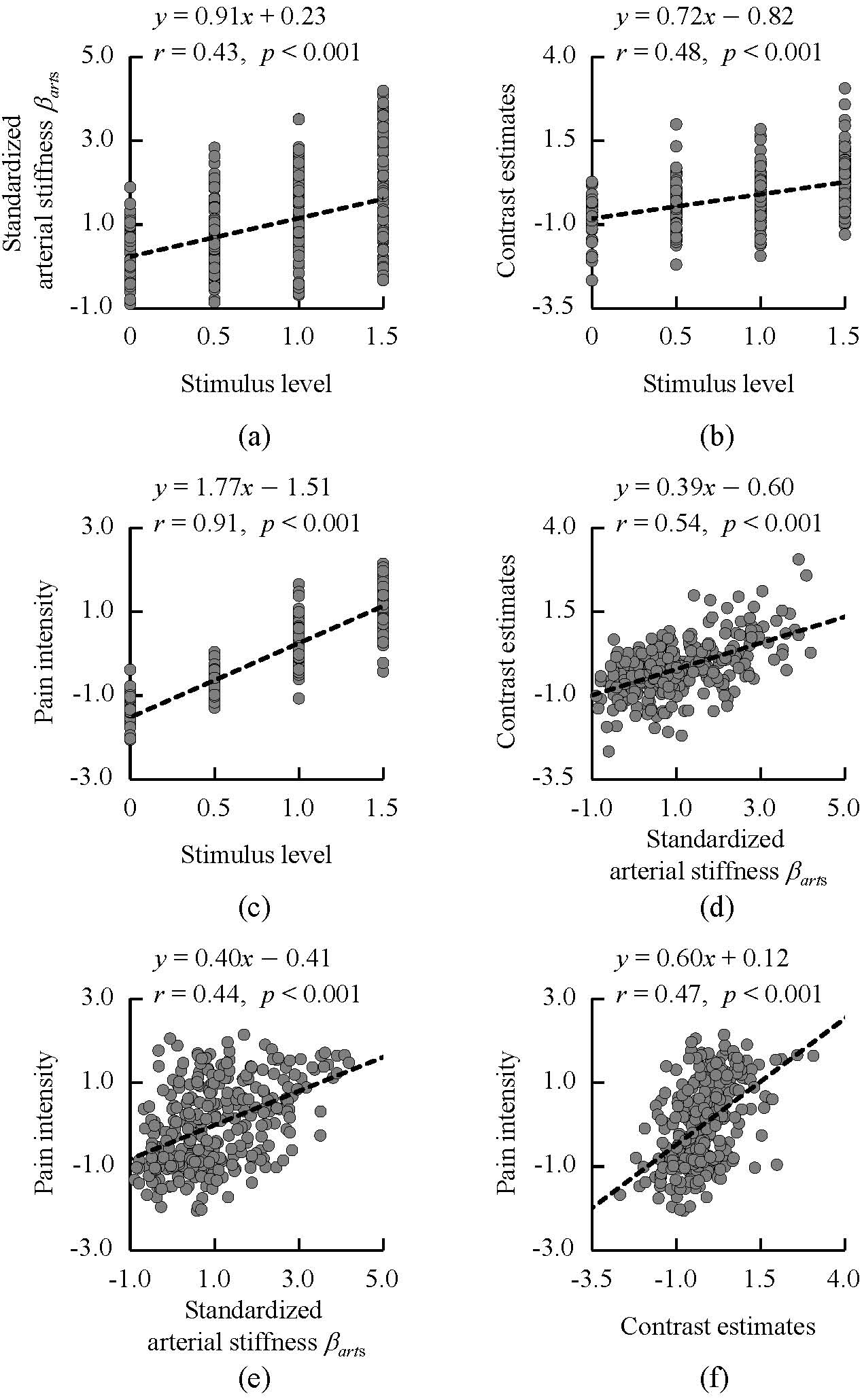


Figure S4-3. Correlations between standardised stimulation amplitude, peripheral arterial stiffness parameter (*β*_art_), contrast estimates averaged across all ROIs, and self-reported pain intensity (VAS scores).
